# Prediction of Metal Additively Manufactured Bead Geometry Using Deep Neural Network

**DOI:** 10.3390/s24196250

**Published:** 2024-09-26

**Authors:** Min Seop So, Mohammad Mahruf Mahdi, Duck Bong Kim, Jong-Ho Shin

**Affiliations:** 1 Department of Industrial Engineering, Chosun University, Gwangju 61452, Republic of Korea; sms3310@chosun.kr; 2 Department of Electrical and Computer Engineering, Tennessee Tech University, Cookeville, TN 38505, USA; mmahdi42@tntech.edu; 3 Department of Manufacturing and Engineering Technology, Tennessee Tech University, Cookeville, TN 38505, USA; dkim@tntech.edu

**Keywords:** wire arc additive manufacturing (WAAM), bead geometry, deep neural network (DNN), gas metal arc welding (GMAW)

## Abstract

Additive Manufacturing (AM) is a pivotal technology for transforming complex geometries with minimal tooling requirements. Among the several AM techniques, Wire Arc Additive Manufacturing (WAAM) is notable for its ability to produce large metal components, which makes it particularly appealing in the aerospace sector. However, precise control of the bead geometry, specifically bead width and height, is essential for maintaining the structural integrity of WAAM-manufactured parts. This paper introduces a methodology using a Deep Neural Network (DNN) model for forecasting the bead geometry in the WAAM process, focusing on gas metal arc welding cold metal transfer (GMAW-CMT) WAAM. This study addresses the challenges of bead geometry prediction by developing a robust predictive framework. Key process parameters, such as the wire travel speed, wire feed rate, and bead dimensions of the previous layer, were monitored using a Coordinate Measuring Machine (CMM) to ensure precision. The collected data were used to train and validate various regression models, including linear regression, ridge regression, regression, polynomial regression (Quadratic and Cubic), Random Forest, and a custom-designed DNN. Among these, the Random Forest and DNN models were particularly effective, with the DNN showing significant accuracy owing to its ability to learn complex nonlinear relationships inherent in the WAAM process. The DNN model architecture consists of multiple hidden layers with varying neuron counts, trained using backpropagation, and optimized using the Adam optimizer. The model achieved mean absolute percentage error (MAPE) values of 0.014% for the width and 0.012% for the height, and root mean squared error (RMSE) values of 0.122 for the width and 0.153 for the height. These results highlight the superior capability of the DNN model in predicting bead geometry compared to other regression models, including the Random Forest and traditional regression techniques. These findings emphasize the potential of deep learning techniques to enhance the accuracy and efficiency of WAAM processes.

## 1. Introduction

In the age of Industry 4.0 and the rise of smart manufacturing, research in additive manufacturing (AM) has seen a significant increase [1]. In 3D printing, AM is an innovative manufacturing technique that constructs physical parts layer-by-layer [2]. Compared to traditional subtractive manufacturing, the greatest advantage of AM lies in its ability to free designers from many manufacturing constraints and eliminate the need for conventional tooling and complex assembly operations [3]. The recent trend in AM has shifted towards the production of high-value metal parts, such as those made from nickel and titanium alloys, particularly in aerospace applications. This shift is driven by the suitability of AM for low-volume production and high customization, addressing the issue of high buy-to-fly ratios of industries [4]. According to ASTM standards, metallic AM processes fall into three main categories: sheet lamination, direct energy deposition (DED), and powder bed fusion (PBF). DED involves the use of either powder feedstock or wire material, along with an energy supply, such as lasers, electron beams, or plasma arcs [5].

Leading the transformation among DED processes is wire arc additive manufacturing (WAAM) which utilizes electric or plasma arcs as the heating element, and metal or alloy wires as the deposition material [1]. In WAAM, a wire is fed into a moving electric arc, which sequentially deposits molten metal according to the path outlined in the computer-aided design (CAD) model. The wire is melted using either a laser source or a welding torch which is controlled by a robot in an inert atmosphere [6]. The WAAM process is distinguished from other metal AM techniques by its flexible manufacturing capabilities, large dimensions, periodicity, significant residual thermal stress, and ability to handle large objects. This flexibility sets it apart from the laser and electron beam AM processes, as the single-layer geometry in WAAM is governed by a short-range free-flow shaping process without a strict correlation between the layer width and heating radius [7]. Among the various WAAM methods, Cold Metal Transfer (CMT) WAAM can produce high-quality spatter-free welded structures with low heat input. The key feature of CMT-WAAM is its ability to control heat input during the welding process. Cold metal transition controls the reciprocating movement of the wire feeding process through digital control and mechanical withdrawal [8]. Despite these benefits, the widespread adoption of WAAM in the industry hinges on improvements in process monitoring and control owing to issues with part quality. The high sensitivity of the electric arcs implies that minor variations in the operating parameters can lead to poor part quality [9].

In the WAAM process, one of the most crucial aspects is the bead geometry, which includes the height and width of the bead. The accurate prediction of bead geometry is essential for controlling the process of producing high-quality parts [10]. The formation of the bead shape is influenced by the interaction of heat and mass transfer, as well as fluid flow, and remains poorly understood. Although improved process parameters can often be identified through extensive experience and numerous trial-and-error experiments, this approach is time consuming and costly. In addition, experiments have struggled to fully capture the complex physical interactions involving the arc, droplet, melt pool, and bead shape, making it difficult to accurately model the WAAM process [11]. To implement an effective WAAM process, understanding the relationship between weld bead geometry and welding parameters is crucial for selecting the optimal welding variables. However, in the current WAAM systems, these variables are often determined manually or with limited automation based on databases and operator experience, which can affect the accuracy and efficiency of the WAAM process [4].

With the advent of artificial intelligence (AI), researchers have begun to use deep learning techniques to analyze the WAAM process in various innovative ways. Numerous modeling methods have been proposed to develop a forward model for weld bead formation, including traditional regression models, the Taguchi approach, and artificial neural networks (ANNs). However, accurately predicting weld bead geometry remains challenging owing to the limited number of welding parameters [12]. By incorporating both process parameters and in-process data comprehensively, machine learning (ML) algorithms such as decision trees (DT), Random Forests (RF), support vector machines (SVM), and extreme gradient boosting (EGB) have been employed to predict bead geometry based on these inputs [4]. Despite these efforts, the non-linear and complex nature of the relationship, along with material property constraints, result in ML models that struggle to achieve high accuracy in predicting bead geometry [13,14]. Alternatively, convolutional neural networks (CNNs) have been used to predict bead geometry using raw images of the weld pool. However, the accuracy of image-based predictions can be compromised by uncontrollable factors during the welding process, such as spattering and reflection from high-intensity arc light on the weld bead surface [15].

Therefore, this study introduces a methodology to predict the bead geometry using a Deep Neural Network (DNN) model under a specific set of process parameters. This predictive model aims to predict and mitigate defects, enabling the proactive control of process parameters and ensuring an optimal bead shape. The effectiveness and practicality of the proposed DNN model were validated experimentally using the CMT-WAAM process. Deep Neural Networks (DNNs) provide flexibility in handling various data types, simple architectures, and high computational efficiency, making them ideal for numerous regression tasks, particularly with smaller datasets. They adapt seamlessly to different regression tasks without requiring spatial invariance, and typically have shorter training times. Furthermore, DNNs are highly customizable, easy to interpret, and effective for both structured and unstructured numerical data that lack inherent spatial dependencies. The remainder of this paper is structured as follows: Section 2 reviews the background and related literature; Section 3 outlines the proposed methodology; Section 4 elaborates on the proposed architecture; Section 5 presents the results and discussion; and Section 6 concludes with remarks and potential future directions.

## 2. Related Works

In this section, the background literature on bead geometry predictions and modeling is reviewed based on prediction mechanisms. The subsequent paragraphs outline the progression from physics-based and statistical models to data-driven approaches such as ML, CNN, and DNN, and the DNN was found to be effective at forecasting.

Statistical and physics-based modeling techniques have dominated the prediction of bead geometry in metal AM. These methods rely on empirical relationships and the fundamental principles of heat transfer and fluid dynamics to forecast bead dimensions and morphology. Geng et al. [7] proposed a central composite rotatable framework to explain the relationships between the wire feed rate, travel rate, and peak current with the bead height and width. The developed model, which was validated by ANOVA, predicted the bead geometry with 95% confidence and advised the optimal parameters for the desired outcomes. Dinovitzer et al. [5] conducted an analysis of TIG-based WAAM using Hastelloy X alloy and 304 stainless steel, employing the Taguchi method and ANOVA to assess the effects of wire feed speed, inert gas flow rate, peak current, and travel speed on bead shape. They found that bead height increased with wire feed rate, bead width decreased with travel speed, and travel speed/current inversely affected heat input. Panda et al. [6] suggested measuring the bead height and width using gene expression programming (GEP) and multigene genetic programming (MGGP) to model these dimensions. Performance analysis via statistical error metrics showed that the GEP models outperformed the MGGP models, with a high $R^{2}$ of up to 0.99. Ma et al. [16] proposed the use of ANOVA and RSM to analyze the effects of travel speed, arc voltage, and arc current on bead dimensions in MIG-based WAAM. They observed that the voltage significantly affected the bead width, whereas the welding velocity predominantly influenced the bead height. Veiga et al. [17] developed a study using GMAW-based WAAM to optimize the conditions for deposition, focusing on the bead symmetry analyzed using symmetry coefficients. They applied symmetry analysis techniques to monitor bead and wall geometry, achieving high symmetry coefficients close to 0.99 and 0.998, respectively, and proposed a melt-pool monitoring algorithm for maintaining symmetry during manufacturing. Chen et al. [3] applied a co-ordinate-transforming recursive overlapping model to forecast multi-bead dimensions in robotic gas metal arc welding (GMAW). They validated the model through experimental tests, demonstrating improved accuracy in describing profile leveling and lower relative errors (1.95–7.06%) compared to conventional methods. Parabolic and arc functions were found to be optimal for predicting DH36 steel profiles. Bernauer et al. [18] suggested a physics-based control architecture for the layer height in WLAM using laser line scanning to obtain 3D part profiles and segmenting weld beads for discrete height control. Their approach effectively compensated for disturbances, ensuring dimensional accuracy without online adjustment of height increments, applicability to various geometries, and scalability to different materials and component sizes. Chen et al. [11] implemented a detailed wire feed model incorporating a novel surface heat source and improved momentum source models into a bead shape. The simulation results matched the experimental data well, identifying three metal transfer modes with varying wire-feeding speeds and analyzing the effects, such as arc shading, on the melt pool dynamics and bead geometry, with errors ranging from 3.9% to 17.1% for the bead width, height, and penetration depth. Although statistical and physics-based models offer foundational insights into bead geometry prediction, their reliance on empirical relationships and simplified physical assumptions can limit their accuracy and adaptability to new and varied metal AM processes. These methods are mathematically intensive and require perfect knowledge of the geometry and melt pool dynamics, which can be challenging. As the complexity of metal AM systems increases, machine learning (ML) approaches present a compelling alternative, leveraging large datasets to capture intricate dependencies that traditional models might miss.

ML approaches have been increasingly applied to predict bead geometries in metal AM processes. Techniques such as SVM and RF utilize training data to infer the relationships between the process parameters and bead characteristics. For example, Wang et al. [8] suggested employing the Box-Behnken method and high-precision laser scanning to explore the effects of process parameters on bead geometry in CMT-WAAM. They developed an ML regression model with less than 5% relative error for the track width and layer height, highlighting the significant influences of feed rate and travel on the bead shape. Ding et al. [4] developed a bead-shaped model featuring an SVM process for high-accuracy model creation. They achieved the optimal wire feed rate and travel speed combinations with mean squared error (MSE) values of 0.0687 for the overlapping distance, and 0.0077 for the bead height. Yaseer et al. [19] predicted surface roughness using the RF algorithm and multilayer perceptron with MSEs of 0.00063 and 0.00087, respectively. Oh et al. [20] proposed SVM and regression learning to achieve an even deposition bead shape in WAAM. The validation showed average width and height errors of 1.28% and 0.36%, respectively. Chandra et al. [21] implemented five ML algorithms to predict the bead geometry in WAAM. The ANN achieved the highest $R^{2}$ value (0.6505) and the lowest MSE (0.0611) and MAE (0.1893) for bead height prediction, while linear regression outperformed the others in bead width prediction with $R^{2}$, MSE, and MAE of 0.8486, 0.1730, and 0.3157, respectively. Chen et al. [22] applied a multisensor fusion-based digital twin for confined quality detection in the L-DED process, achieving a defect prediction accuracy of 96%. They employed ML models, including SVM, KNN, DT, RF, NN, linear regression (LR), and GB, with optimal hyperparameters to generate a virtual bead-shaped map, reducing the need for physical inspections. Gihr et al. [23] performed WAAM geometry prediction for multi-layer bead depositions using an MLP model, achieving an $R^{2}$ error of 0.7 for bead shape prediction. Their ML approach outperformed traditional analytical methods by considering dynamic material flow, and suggested optimal welding parameters for smooth and uniform surfaces. He et al. implemented an advanced algorithm combining ANFIS and PSO to learn welding skills from proficient human welders and achieved root mean squared error (RMSE) values of 0.1648 and 0.1805, respectively, for fabricating WAAM parts. The model accurately generated continuous deposition parameters and outperformed traditional methods by closely matching the target geometries [24]. Although ML algorithms such as SVM and RF enhance predictive capabilities by learning from data, they often struggle with the high-dimensional and spatially complex information inherent in metal AM bead geometry. However, these methods lack automatic feature extraction, exhibit poor pattern recognition, and handle noise and variability inadequately. CNNs address these limitations through superior data processing and learning from spatially structured data, and are suitable for predicting bead geometries with high accuracy and robustness.

CNNs transform the prediction of the bead geometry by leveraging their ability to learn spatial features from imaging data. In metal AM, CNNs capture complex patterns through a layer-by-layer deposition process. Jamnikar et al. [25] proposed a CNN model for real-time monitoring of WLAM molten pool data and predicted geometric parameters (area, width, height, and depth) with RMSE values of 3.39 mm^2^, 0.5 mm, 0.3 mm, and 0.9 mm, respectively. Their model achieved a prediction error of less than 10% for all the parameters. Alexander et al. [9] implemented SVR and CNN models for real-time process control, demonstrating their ability to accurately predict the bead height from melt pool images captured by a coaxial optical camera. Both models achieved a mean absolute percentage error (MAPE) of approximately 3.67%, highlighting their effectiveness in recognizing complex non-linear patterns in additive manufacturing. Mochi et al. [26] developed a machine learning model using transfer learning with VGG, ResNet, and DenseNet CNN architectures for real-time prediction of bead widths using melt pool images. The model achieved an MAE of 4.5%, with VGG delivering a similar performance. Dong et al. [14] developed a multimode convolutional neural network (M-CNN) to predict the deposited layer sizes (width, height, and cross-sectional area). The M-CNN, integrating ResNet18 and an FCNN, achieved a low mean squared error (MSE) of 0.016, 0.03, and 0.054 for the width, height, and area, respectively, outperforming the SVR and RF baselines in predicting layer dimensions. Wang et al. [10] proposed a physics-motivated temporal convolutional network (TCN) approach for high-accuracy predictions in LDED processes. The TCN model integrates the physics of the highest temperature, Marangoni effect, and molten jet to enhance the prediction accuracy. The experimental validation of the thin-walled parts demonstrated MAPEs of 3.421% for the bead width and 4.643% for the bead height prediction. Baek et al. [15] implemented a deep learning method to detect the melt-pool depth using melt pool images. A residual neural network was employed for segmentation of the melt pool shape, followed by backpropagation for regression to predict the depth. The model achieved an MAE of 0.0596 and $R^{2}$ of 0.9974 in experiments using gas tungsten arc welding (GTAW). Asadi et al. [27] developed a real-time monitoring approach for L-DED using CNN-based models to segment bead images and predict key parameters such as bead shape and area. YOLOv8 models, particularly YOLOv8l, demonstrated high accuracy with mean average precision (mAP) values of 0.925 and 0.853 for stainless steel and low-carbon steel substrates, respectively, and achieved processing speeds exceeding 114 frames per second, highlighting their efficacy for real-time process control. Nikolaou et al. [28] developed a two-scale approach using micro-CT and YOLO v7 to segment fibers and voids in 3D-printed composite filaments. Despite the strengths of CNNs in handling spatial data, their architecture is primarily optimized for image-based tasks and may not fully capture the multifaceted relationships between metal AM process parameters and bead geometry. Moreover, CNNs are inflexible in terms of the data type, and entail complex architectures that require more computation time. DNNs, with their deeper and more flexible architectures, provide a more comprehensive approach for modeling complex non-linear interactions. They are particularly effective for small datasets, are adaptable to diverse regression tasks, and have shorter training times than CNNs because of the absence of convolutional layers, which speed up bidirectional propagation processes, thus offering improved predictive performance in diverse AM scenarios.

DNNs have emerged as powerful tools for predicting bead geometries in metal AM. Their hierarchical layers enable them to learn intricate mappings between the process parameters and geometric outcomes, surpassing traditional linear models. For example, Glasder et al. [29] applied a novel framework for accurate weld bead stacking prediction, dividing it into footprint and shape prediction tasks using deep convolutional neural networks and a surface energy minimization model. Their approach achieved median accuracies of 0.1 mm for bead width prediction and 0.19 mm for bead length prediction. Mu et al. [1] proposed an adaptive in situ simulation using a VQVAE-GAN and RNN architecture to predict the distortion fields and bead geometry in AM-DT. Pretrained with FEM simulations, it achieved superior performance with an RMSE under 0.9 mm, outstripping FEM by 143% and ANN by 151%, as validated on seven thin-wall structures. Nam et al. [12] developed a DNN monitoring system using the VoVNet27-slim architecture to predict bead geometry during WLAM. The two-image model achieved a validation MAE of 0.0218 and test MAE of 0.02123. Wei et al. [2] developed a DNN-augmented architecture combining a physics-based predictor with a DNN-based error detector to forecast the bead roughness in fused deposition modeling. The model achieved MAPE values of approximately 3.15%. Cooper et al. [13] implemented an adversarial network namely CGAN to analyze the power indicators for augmenting data in a DNN-based bead roughness detection framework. Using this approach, prediction error reduced from 58% to 9.1% for 250 samples. Oh et al. [30] applied a DNN algorithm for bead geometry measurements in GMAW welding, achieving over 96% accuracy in predicting the bead width and height. Using real-time current and voltage waveforms as the input data, the DNN was trained and evaluated using the predictive ability of the model (PAM), with MSEs of 0.1141 during training and 0.1536 during testing. DNNs offer substantial improvements over previous predictive methods for the bead geometry in metal AM. Unlike statistical and physics-based models that require extensive mathematical knowledge and a perfect understanding of geometry and melt-pool dynamics, DNNs can learn complex non-linear relationships from data. Compared to traditional machine learning methods, which lack automatic feature extraction and struggle with noise and variability, DNNs excel at capturing intricate patterns. Although CNNs are limited by their inflexible data types and complex architectures, which increase computation time, DNNs are highly adaptable to diverse regression tasks and are effective for small datasets, which are common in specialized AM applications. They also benefit from shorter training times owing to the absence of convolutional layers, which accelerate the forward and backward propagations. These advantages led us to develop a DNN architecture for bead geometry prediction, aiming to enhance the accuracy and quality of metal AM processes by leveraging their adaptability, efficiency, and superior performance.

## 3. Proposed Methodology

**Figure 1:**
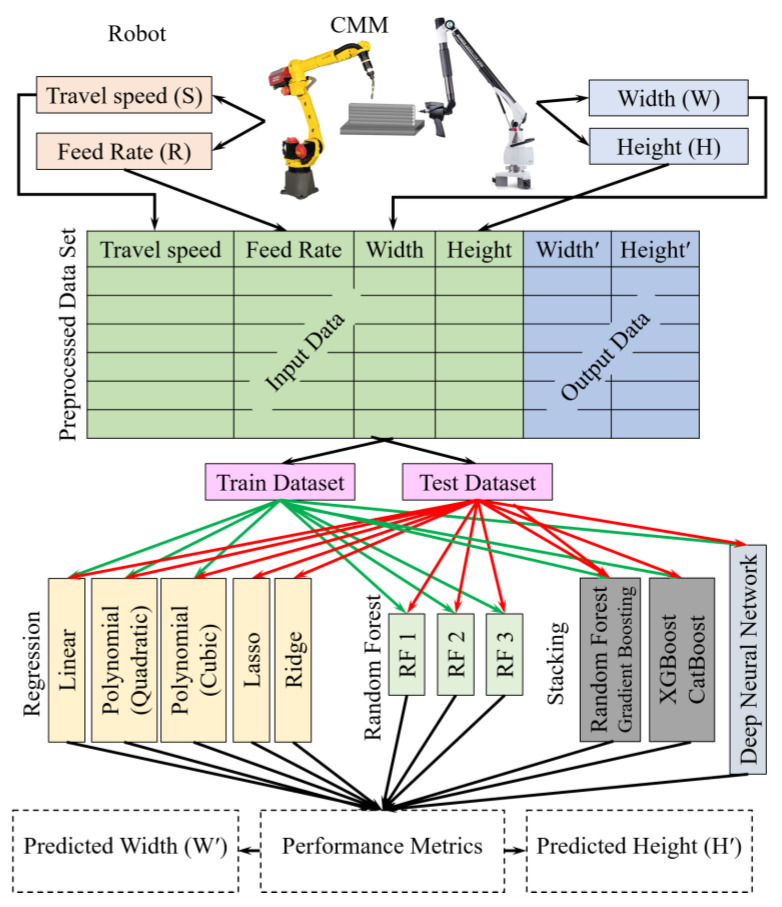
Proposed methodology for implementation of the Deep Neural Network (DNN) model.

The aim of this study is to build a robust predictive framework for bead geometry in metal AM, concentrating on bead width and height, which are critical for ensuring the structural quality of the manufactured parts. As shown in Figure 1, the methodology involves collecting input data comprising the travel speed (S), feed rate (R), and bead width and height of the previous layer, which are monitored using a system integrated with a co-ordinate measuring machine (CMM) to ensure precise measurements. The collected data were organized into training and testing datasets, which are used to develop and validate various regression models including Linear, Ridge, Lasso, Polynomial (Quadratic and Cubic), RF, and the proposed DNN. Among these models, the RF model and custom-designed DNN are particularly notable. As part of an ensemble learning technique, the RF model builds several decision trees during training and delivers an average prediction, which lowers overfitting and boosts generalization. Additionally, the DNN model, specifically tailored for this task, comprises multiple hidden layers and neurons trained through backpropagation to minimize the RMSE between the original and predicted bead geometries.

**Figure 2:**
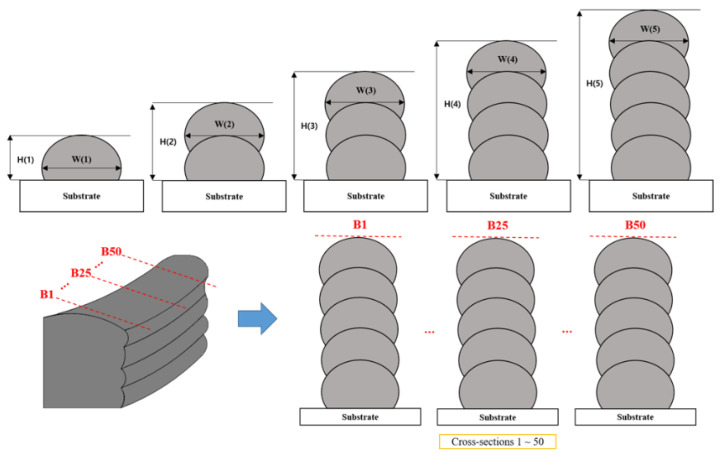
Definition and calculation of bead width (W) and bead height (H).

The prediction of the bead geometry begins by precisely defining the width and height, as shown in Figure 2. The width (W) of a bead refers to the widest distance between adjacent layers, which is essentially the maximum horizontal span between two layers when viewed from the side. In practical terms, W dictates the amount of material deposited in each pass, where a wider bead may enhance bonding but risks the accumulation of excess material. Height (H) is represented by the collective length from the posterior of the initial layer to the zenith of each succeeding layer, with each layer incrementally contributing to the total height. H influences the overall strength, surface finish, and structural integrity of the manufactured part, making its control crucial for obtaining consistent results. Traditionally, bead shape determination relies on analyzing a single cross-section of the wall, which is a method with inherent limitations. A single cross-section may not accurately represent the characteristics of the entire wall, particularly if there are variations within the bead. To overcome this limitation, multiple cross-sections are created by dividing the point clouds of the surface profile into regular intervals (50 regular intervals). Each interval provides a cross-sectional view of the beads. Analyzing these cross-sections collectively yields a more comprehensive understanding of the bead shape. The average bead shape derived from all the sections signifies the bead geometry for the entire area of each thin wall. By averaging the variations observed across different cross sections, a more accurate representation of the bead geometry was obtained. In summary, precise bead geometry involves considering both the width and height, overcoming the limitations of single-cross-sectional analysis, and arriving at an average shape. This approach enhances the predictability and quality of additive manufacturing processes.

In the process of measuring the bead geometry, a CMM involves the capturing of precise measurements of the bead’s height and width using point-cloud data. The CMM scans the additively manufactured wall, generating three-dimensional co-ordinates (x, y, z) that represent various points along the surface profile. These detailed data are crucial for analyzing the bead geometry. The point clouds provided a comprehensive map of the wall surface, from which the width and height were identified. By dividing the surface profile into regular intervals, each interval yields a cross-sectional view, facilitating the identification of the geometric properties of the bead across the entire structure. This method ensures that even minute variations in bead shape are captured, leading to accurate and reliable measurements. The point-cloud technique, with its high-resolution data, allows for a thorough analysis of bead geometry, enhancing precision and quality control in metal additive manufacturing processes.

### 3.1. Experimental Setup

**Figure 3:**
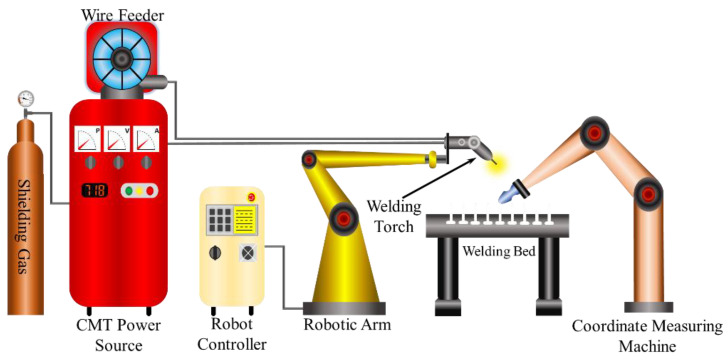
Experimental setup of the GMAW-CMT WAAM process.

The experimental framework illustrated in Figure 3 employs three primary systems, one each for movement, material deposition, and measurement. The movement apparatus was a robotic manipulator (Fanuc ArcMate 120iC (FANUC Corporation, Rochester Hills, MI, USA)) that finely controlled the positioning during the deposition phase. Previous research on WAAM indicates that the bead shape is heavily influenced by the thermal input, particularly at high deposition speeds. To address these thermal effects, Cold Metal Transfer (CMT) technology was developed by the Austrian company Fronius. CMT is highly regarded for its ability to generate minimal heat input and ensure high arc stability, thanks to an innovative wire feeding system combined with a digital control setup [31]. Due to these advantages, the experiment utilizes CMT technology, integrating a Fronius TPS 400i welding power source with a Fronius WF 25i Robacta Drive (Fronius International GmbH, Wels, Austria) welding torch, employing the GMAW-CMT process. This process is widely used in the aerospace, automotive, and marine industries for its effectiveness in managing high-speed deposition with minimal defects, ensuring consistent bead quality and reducing heat-related issues [32]. The deposition and movement parameters were meticulously controlled using the respective system controllers. Additionally, the geometric profile of the layered wall was measured using a CMM; specifically, a Hexagon Romer Arm 7525SIE (Hexagon Manufacturing Intelligence, North Kingstown, RI, USA) model was used to capture three-dimensional point clouds for detailed analysis.

Using the GMAW-CMT process, a wall was constructed on a stainless-steel 316 L substrate with dimensions of 6 × 2 × 0.25 inches. The wire material used for the deposition was also stainless steel 316 L, ensuring material consistency. To minimize the distortion during the welding process, the substrate was securely clamped on both sides to prevent movement. The optimal bead geometry is achieved with a deposition angle between 20° and 60°. It has been reported that angles greater than 60° cause severe rippling on the surface, while angles below 20° lead to instability in the bead formation [33]. Therefore, the wire feed angle was set at 30° towards the interior from the top surface to optimize bead formation. A consistent deposition direction throughout the process ensures uniform bead characteristics. Despite the precision offered by the robotic arms, inherent positioning accuracy issues persist. However, this study focuses on controlling the layer geometry at the macro level, thus, processing errors owing to robot arm movements are not considered critical for the scope of this research.

### 3.2. Data Collection and Preprocessing

Enhancing process planning and deposition strategies in AM is crucial for achieving an optimal combination of parameters. To accomplish this, it is necessary to correctly set the process parameters such as the wire feed rate and travel speed. Predicting the bead geometry based on the selection of process parameters is essential for determining near-optimal settings. Each selected parameter was then evaluated and compared to identify the best process configuration. This predictive modeling approach for bead geometry can significantly influence the selection and optimization of process parameters, ultimately refining the surface quality of the produced part. The specifications of the GMAW-CMT WAAM process are listed in Table 1. The process parameters, which are adjusted for the deposition of each new layer, include the travel speed (adjustable from 1 to 12,000 cm/min in one-unit increments), and feed rate (customizable from 100 to 1000 cm/min in 10-unit steps). The static parameters, which remained constant throughout the process, included the previous layer temperature set at 100 °C to mitigate heat effects, an arc length of 5 mm, a wire diameter of 1.2 mm, a wire feeding angle of 30°, and a shielding gas composition of 100% with a flow rate of 20 L/min.

Using the GMAW-CMT technology, 27 thin walls of five layers each were created to gather experimental data on dynamic process parameters and bead shape. As shown in Table 2, the dynamic process parameters, including feed rate and travel speed, were selected based on prior research findings, which demonstrated their significant impact on bead geometry in WAAM processes [34,35]. Specifically, three combinations of these parameters were established to deposit the layers, as they have been shown to directly influence bead width, height, and overall surface roughness.

The measured bead shape and dynamic process parameters were used to define the input and output data for model training in the following step. The bead shape is typically evaluated by examining a single wall cross-section, which cannot accurately reflect the features of the entire wall. To overcome this restriction, surface profile point clouds of each wall were periodically split into 50 cross-sections. Subsequently, a smoothing procedure transforms the surface profile of each cross section, which is expressed by point clouds, into a single line. The bead shape of every cross-section is measured, and the average bead shape of the overall cross-sections is selected to reflect the overall bead form of the wall. This comprehensive method guarantees that the observed bead shape, dynamic process parameters, and general bead geometry are appropriately represented in the input and output data required for model training.

## 4. Model Development

### 4.1. Definition of Input Data and Output Data

The development of a DNN model is fundamentally dependent on the definition of the input (features) and output data (labels/targets). The input data consist of varying process parameters and the bead geometry of the previously deposited layer, whereas the output data include the bead geometry of the subsequent layer. Table 3 presents the structures of the input and output data used in the model. The input data feature parameters include the width and height of the current layer bead, travel speed, and feed rate. These dynamic parameters, which influence the deposition process, are reset for each new layer. The output data, on the other hand, represent the width and height of the bead in the next layer, reflecting the predicted geometry based on the input parameters provided. This approach ensures that the model effectively captures the relationships between the process parameters and resulting bead geometries, which is crucial for precise predictions.

‘Index’ and ‘Number of Thin Walls’, the first two columns, show the layers that have been processed in each wall, in order. Each wall consists of multiple layers, each of which contains data captured in the columns that follow it from the current layer. These columns document the dynamic process parameters for the current layer, as well as the width and height of the bead. The expected bead height and width of the following layers are shown in the final two columns: model output or target. Normalization is applied to every variable to minimize deviations from various measurement scales and handle varying value ranges. This method helps avoid accuracy loss from large computations and shortens the learning curve of machine learning models. A robust scaler normalization approach is employed in this study to guarantee that the data are appropriately scaled for the best model performance.

A systematic approach is employed to prepare the dataset for training and testing the DNN model for predicting bead geometry in metal additive manufacturing. The process begins with data loading and preprocessing, in which the process parameters and bead shapes of the previous layers are extracted as features. The next step involves seeding and splitting the dataset to ensure the replicability and reliability of the results. Seeding using a random seed value ensures that the random selection process is consistent every time code is run. This allows the same set of indices to be selected, ensuring replicability of the results. By setting a seed value (i.e., np.random.seed in (42)), the random number generator produces the same sequence of random numbers, which means that the same subsets of data are used for training and testing in every run of the code.

After setting the seeds in the coding script, random indices are generated to divide the data into training (80%) and testing (20%) sets. Specifically, 20 random indices are chosen from a range of 0 to 26. Each selected index corresponds to a range of rows (i.e., four consecutive rows) in the dataset. These rows are then extracted and concatenated to form the training dataset (features and targets). The features considered in this case are travel speed (S), wire feed rate (R), width of previous layer (W), and height of previous layer (H), whereas the labels or targets were the next layer width (W’) and next layer height (H’). The remaining rows, which have not been included in the training set, are used for the testing set (features _test and target_test). This approach ensures that the training set contains diverse examples from different parts of the dataset, thereby enhancing the ability of the model to generalize. The final step in data preparation involved converting the training and testing datasets into Numpy arrays and saving them for future use. The feature array includes features such as the current layer bead shape and dynamic process parameters, whereas the target array includes the bead shape of the next layer. The testing datasets (features _test and target_test) exhibited the same structure. This structured and repeatable approach to data splitting ensures that the model can be consistently trained and tested, thereby providing reliable and comparable results across different experiments.

### 4.2. DNN Model Development

**Figure 4:**
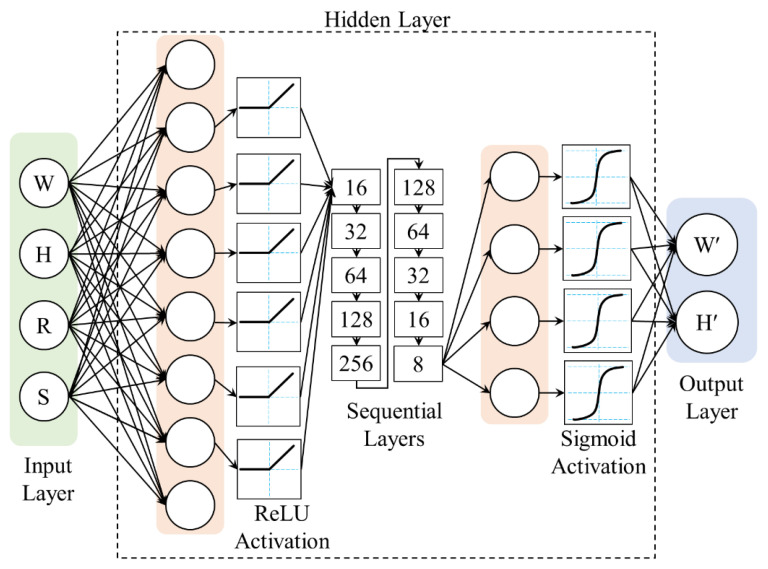
Development of the proposed DNN model.

DNNs are employed to predict the bead geometry with high accuracy. Their architecture processes and abstracts high-level data features, rendering DNNs particularly effective for identifying patterns within datasets. The geometry of the bead, which is influenced by various factors, requires a model capable of learning from these complexities. The DNN achieves this by channeling the input through successive layers, each refining the data further. This process enables the network to gradually identify and learn patterns, culminating in predictions from the output layer. The network weights are finely tuned through iterative backpropagation and optimized using methods such as gradient descent. The architecture of the DNN model is shown in Figure 4, illustrating its capability to uncover data patterns associated with the bead geometry.

The DNN model was constructed using PyTorch (version 2.4.1) and trained in a Google CoLab environment. The training was conducted using an NVIDIA Tesla T4 GPU, which provided approximately 16 GB of GPU memory. The Colab environment was configured with 12 GB of RAM and a Python 3.x environment. The model architecture was initialized using the Kaiming normal method to ensure proper weight scaling and stability during training. The network includes an input layer that combines feature dimensions, followed by multiple hidden layers with rectified linear unit (ReLU) activation functions, and an output layer that predicts the bead width and height with sigmoid activation. The structure of the model is defined as follows. The input layer accepts a combined feature vector, followed by hidden layers with neuron counts of 8, 16, 32, 64, 128, and 256, and then symmetrically decreases to two in the output layer. This hierarchical structure allows the network to capture complex non-linear relationships in the data.

The complex non-linear relationships that the DNN captures include interactions between various process parameters and the resulting bead geometry. For instance, the bead width and height are influenced by multiple factors such as travel speed, feed rate, and the characteristics of the previous layer. These factors do not have a straightforward linear relationship with the bead geometry. Instead, the effects of changing one parameter can vary depending on the values of other parameters. For instance, an increase in travel speed could reduce the bead height when the feed rate is low, but may have a different effect when the feed rate is high. Additionally, the shape of the previously deposited layer influences the geometry of the current layer in a non-linear fashion. These interactions create a complex web of dependencies that are challenging to model using linear approaches. A DNN, with its multiple layers and non-linear activation functions, can learn these intricate relationships by transforming the input data through successive layers, each capturing different aspects of the data structure.

The forward propagation mechanism involves sequentially passing input data through the network layers. Each layer applies linear transformations followed by ReLU activation functions except for the output layer, which outputs the predicted bead width and bead height. The data flow through the network as follows. Input features are first processed by the initial hidden layer, where weights and biases are applied and adjusted through activation functions, producing an intermediate output. This output is then passed to the next hidden layer, where the process is repeated, enabling the network to build increasingly complex representations of the input data. Backward propagation is the process of optimizing the network weights to minimize prediction errors. Network performance is assessed using a loss function, namely the L1 loss in this model, which measures the absolute difference between the predicted and actual bead geometries. The choice of the L1 loss is motivated by its robustness to outliers, as it does not square the error terms, unlike the L2 loss (Mean Squared Error). This makes L1 loss particularly effective in scenarios where the data may contain outliers or noise, as it minimizes their impact on the overall model performance. The Adam optimizer is employed to adjust the network weights using gradients computed from the loss function. During backpropagation, the optimizer updates the weights in each layer and moves them in a direction that reduces loss. This iterative process continues until the model converges to a state in which the predictions closely match the actual bead geometries, ensuring that the network accurately captures the relationships in the input data.

### 4.3. Random Forest, Regression, and Stacking

To perform a comparison with the base ML models, Random Forest, Regression, and Stacking techniques were implemented, which provided a comprehensive evaluation of the different modeling approaches for predicting the geometry of additively manufactured beads, as opposed to the proposed DNN model.

- Random Forest

**Figure 5:**
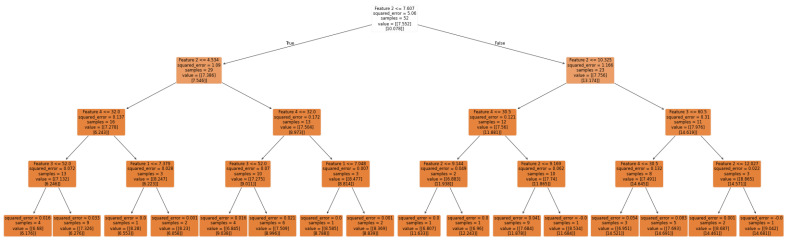
Development of best parameters Random Forest model.

The RF model was used to predict the geometry of the additively manufactured beads. Initially, various parameter configurations were tested to optimize the performance of the model. These configurations involved adjusting the number of trees (n_estimators), the minimum number of samples required to split a node (min_samples_split), the minimum number of samples required at each leaf node (min_samples_leaf), and the maximum tree depth (max_depth). The configurations included 200 trees with a maximum depth of 8 and a minimum of eight samples for both splits and leaves; 100 trees with a maximum depth of 4 and a minimum of eight samples for both splits and leaves; and 100 trees with a maximum depth of 2 and a minimum of 2, a minimum of 4 samples at each leaf, and 8 samples to split a node, as shown in Figure 5. Each configuration was used to train the model on the state and action features, and predictions were made for the test set. The performance of the model was evaluated using metrics such as MAPE, RMSE, and the Pearson correlation coefficient (r) to compare the predicted bead geometry with the actual measurements. Additionally, R-squared ($R^{2}$) values were calculated to assess the proportion of variance in the dependent variable that was predictable from the independent variables.

GridSearchCV was used for hyperparameter tuning to further refine the RF model. The grid search process involved exploring a range of values for each hyperparameter: n_estimators (100, 200, 300), max_depth (4, 6, 8, none), min_samples_split (2, 4, 8), and min_samples_leaf (1, 2, 4, 8). This exhaustive search across the parameter grid aimed to identify a combination that minimized the mean-squared error in a five-fold cross-validation. After identifying the optimal hyperparameters using GridSearchCV, the best RF model, as shown in Figure 5, was retrained using the entire training dataset, and then evaluated on the test set.

- Regression Model

**Figure 6:**
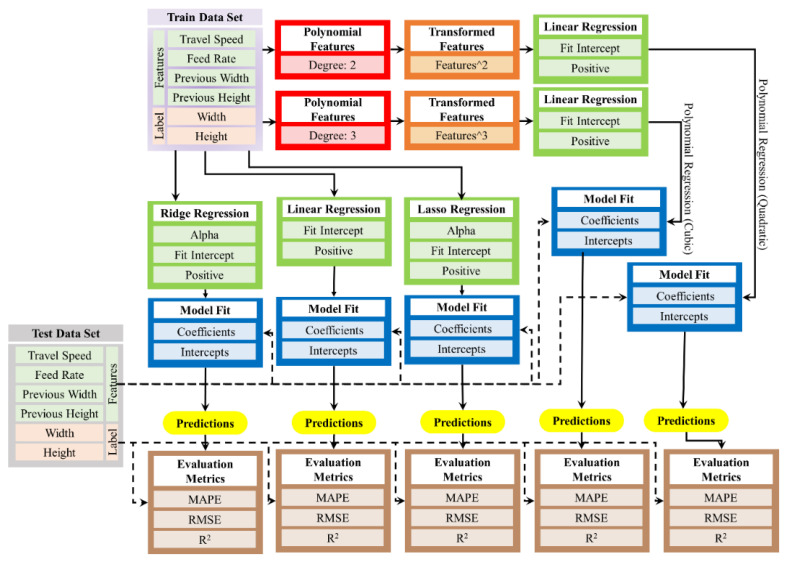
Implementation of regression models.

In addition to the Random Forest model, several regression models were implemented to predict the bead geometry. These included Linear Regression, Ridge Regression, Lasso Regression, and Polynomial Regressions of degree two (quadratic) and degree three (cubic). Each regression model was evaluated using the same test set and metrics as those used in the RF model. The process is depicted in a structured diagram in Figure 6, detailing each step from data preparation to model evaluation. The methodology begins with data preparation, in which both a training dataset and a test dataset are created. The training dataset comprises input features such as travel speed, feed rate, previous width, and previous height, with width and height as output labels. The same set of features was used to prepare the test dataset to ensure consistency in the data used for the model evaluation.

Next, the features were subjected to polynomial transformations to enhance the ability of the model to capture non-linear relationships. Two polynomial feature transformations were applied: one up to degree 2 and the other up to degree 3. This step is crucial for enriching the feature set and potentially improving the predictive power of the regression models. The methodology then progresses to the application of various regression models. Linear regression is implemented with configurations to fit the intercept and, optionally, to constrain the coefficients to be positive. This model is applied to both the original and polynomial-transformed features. Additionally, Ridge regression and LASSO regression are employed, both incorporating the tuning of the alpha parameter, fitting of the intercept, and positive constraints. These regularization techniques help manage multicollinearity and enhance model generalization. Once the models are configured, they are fitted to the training dataset to generate coefficients and intercepts representing the fitted parameters. The trained models are then used to predict bead geometry (width and height) using a test dataset. The predictions from each model are evaluated using key metrics that provide a quantitative measure of the model’s predictive accuracy and overall performance.

Linear Regression was implemented with a parameter grid for hyperparameter tuning using GridSearchCV, including options for fitting the intercept and constraining the coefficients to be positive. The Ridge Regression model introduced a regularization term controlled by the alpha parameter, which was tuned using the GridSearch CV. Similarly, the Lasso Regression, which employs L1 regularization, was tuned to determine the optimal alpha value by fitting the intercept and positivity constraints. For polynomial regression, a feature transformation was performed to include polynomial features up to a specified degree. Quadratic regression involves transforming the state and action features to include all polynomial combinations up to degree two, while cubic regression includes all combinations up to degree three. These transformed features were then used to fit linear models using GridSearchCV to determine the best hyperparameters.

- Stacking Model

**Figure 7:**
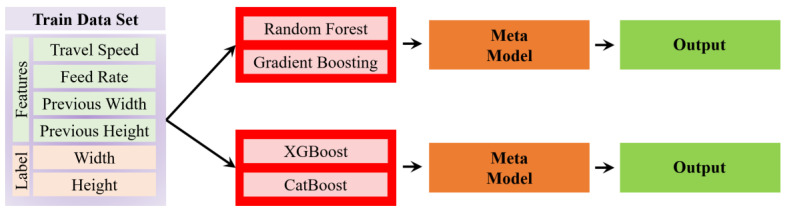
Implementation of stacking models.

In addition to other models, a stacking ensemble model was implemented to further improve the predictive accuracy of the bead geometry. Stacking is an advanced ensemble technique that combines the predictions of multiple base models to produce a more accurate and robust final prediction by leveraging the strengths of each model, while mitigating their individual weaknesses. Two base-model configurations were tested within the stacking framework. The first configuration utilized Random Forest and Gradient Boosting as the base models, which were chosen for their strong performance in handling non-linear relationships and variance. The second configuration employed XGBoost and CatBoost, both of which are known for their efficient handling of large datasets and ability to model complex interactions (See Figure 7). Each base model was trained on the same input features, including travel speed, feed rate, previous width, and previous height. The predictions generated by these base models were combined using a Linear Regression model as the metamodel. This metamodel effectively synthesizes the outputs from the base models, making adjustments to improve the final prediction accuracy.

The performance of the Stacking models was evaluated using the same key metrics as the individual models, including MAPE, RMSE, and Pearson correlation coefficient, providing a comprehensive assessment of the model’s predictive accuracy and overall performance.

## 5. Result and Discussion

This section of the paper provides a comprehensive analysis of the predictive performance of different models for bead geometry in metal additive manufacturing. We employed the proposed DNN, Random Forest, and the following regression models: Linear Regression, Polynomial Regression (Quadratic and Cubic), Ridge Regression, and Lasso Regression. These models were chosen for their different approaches to handling multicollinearity and feature selection, which are critical for capturing complex relationships within the data. The evaluation of these models is based on key metrics: MAPE, RMSE, and R-squared ($R^{2}$). These metrics were selected to provide a holistic view of the model accuracy and reliability. We compared the patterns of the original and predicted data, focusing on the $R^{2}$ analysis, to quantify how well the models explained the variance in the bead geometry. By examining these aspects, we aimed to identify the most effective model for predicting the bead geometry and facilitating improved control in metal additive manufacturing processes.

The evaluation metrics used in this study are essential for quantifying the prediction performance and understanding the accuracy and reliability of the regression models. Mean Absolute Percentage Error (MAPE) is defined in Equation (1):

(1) $$MAPE=\frac{100\%}{n}\sum_{t=1}^{\;\;n}\left|\frac{y_{i}-{\hat{y}}_{i}}{y_{i}}\right|$$

where $y_{i}$ and $\hat{y_{i}}$ represent the actual and predicted values, respectively, and n denotes the total number of observations. The MAPE measures the prediction accuracy as a percentage, making it easy to interpret the error magnitude relative to the actual values. This is particularly useful for comparing the performance of models across different datasets or units of measurement. A lower MAPE value indicates a higher accuracy, with the model’s predictions closely aligned with the actual values. Second, Root Mean Square Error (RMSE) is another critical metric, defined by the Equation (2):

(2) $$RMSE=\sqrt{\frac{1}{n}\sum_{t=1}^{\;\;n}{\left(y_{i}-{\hat{y}}_{i}\right)}^{2}}$$

The RMSE provides a measure of the average magnitude of the prediction errors. Unlike the MAPE, the RMSE penalizes larger errors more heavily, thereby making it more sensitive to outliers. This metric is beneficial when large errors are particularly undesirable because it highlights the ability of the model to predict precision. A lower RMSE value signifies a better predictive performance, indicating that the model’s predictions are close to the actual observations. The coefficient of determination ($R^{2}$) was calculated using Equation (3).

(3) $$R^{2}=1-\frac{\sum_{i=1}^{n}{\left(y_{i}-{\hat{y}}_{i}\right)}^{2}}{\sum_{i=1}^{n}{\left(y_{i}-\bar{y}\right)}^{2}}$$

where $\bar{y}$ is the mean of the actual values. $R^{2}$ measures the proportion of variance in the dependent variable that is predictable from the independent variables. It ranges from 0 to 1, with higher values indicating a better fit. An $R^{2}$ value close to 1 suggests that the model explains a significant portion of the variance in the response variable, demonstrating a strong predictive capability. Conversely, an $R^{2}$ value close to 0.0 implies that the model failed to capture the underlying patterns in the data. By combining these metrics, we can comprehensively evaluate the performance of the regression models, ensuring robust and reliable predictions in the context of metal additive manufacturing.

Table 4 presents a comparative analysis of the various models used to predict bead geometry in terms of width and height, focusing on metrics such as MAPE, RMSE, and R-squared ($R^{2}$). The results indicate that the DNN model performs poorly, especially for height prediction, compared to the other models, with MAPE values of 0.014 for width, 0.012 for height, and RMSE values of 0.122 and 0.153, respectively.

**Figure 8:**
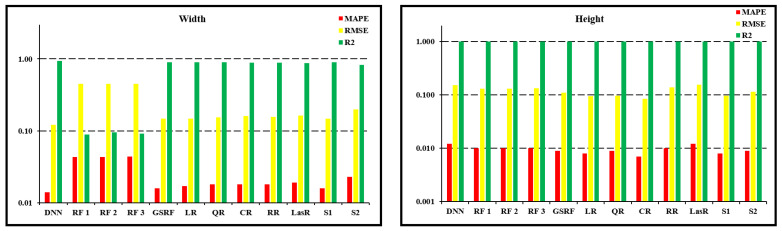
Comparison of performance of the models implemented.

In comparison, the RF models, including RF1, RF2, and RF3, achieved low MAPE values of 0.043–0.044 for width and 0.010 for height, suggesting good accuracy. However, the RMSE values for these models were relatively high (approximately 0.449–0.450 for width and 0.130–0.132 for height), and the $R^{2}$ values were notably lower for width (0.089–0.095), as shown in Figure 8, indicating that these models might not capture the underlying patterns in the data effectively. The Grid-Search Random Forest (GSRF) model showed improved performance, with MAPE values of 0.016 for width and 0.009 for height, RMSE values of 0.148 and 0.109, and $R^{2}$ values of 0.901 and 0.999 for width and height, respectively.

Additionally, the first Stacking configuration, which utilized Random Forest and Gradient Boosting as base models, achieved MAPE values of 0.016 for width and 0.008 for height, RMSE values of 0.148 for width and 0.096 for height, and $R^{2}$ values of 0.90 for width and 0.99 for height. This model performed competitively with the GSRF model, demonstrating strong predictive accuracy, particularly for height prediction. The second stacking configuration, employing XGBoost and CatBoost as the base models, resulted in MAPE values of 0.023 for width and 0.009 for height, RMSE values of 0.199 for width and 0.114 for height, and $R^{2}$ values of 0.82 for width and 0.99 for height. Although this configuration showed slightly lower accuracy in width prediction than the first configuration and the GSRF, it maintained a strong performance in height prediction.

Polynomial regression models, including Linear Regression (LR), Quadratic Regression (QR), and Cubic Regression (CR), show varying performance levels. While the LR and QR models provided low MAPE and RMSE values, the CR model slightly improved the results for height prediction, with a MAPE of 0.007 and an RMSE of 0.084. However, these models generally show strong performance, with high $R^{2}$ values close to 0.999 for height, suggesting that polynomial regression can adequately capture data complexity.

The DNN model, with its specific architecture and hyperparameters, including the ReLU activation function, a layered structure ranging from 2 to 256 nodes, He initialization, Adam optimizer, a learning rate of 1 × 10^−4^, and 10,000 epochs, achieved the most accurate predictions. Although the MAPE values for height appear higher than those of the other models, the R-squared ($R^{2}$) values of the DNN model which were 0.93 for width and 0.99 for height indicate a strong correlation between the predicted and actual bead geometries, suggesting that the DNN model effectively captures the underlying patterns of the data. A higher $R^{2}$ illustrates a residual error between the actual and predicted bead geometries, thereby emphasizing the superior performance of the DNN model. This analysis underscores the importance of selecting appropriate model architectures and tuning hyperparameters to achieve optimal predictive accuracy in additive manufacturing processes. From a cursory observation, the results underscore the robustness and reliability of the RF and regression models in predicting the bead width over the DNN model, which appears to struggle with the given data. However, in reality, the RF and regression models are highly overfitted on the training data, which can be substantiated by considering $R^{2}$ and the data pattern adherence, as detailed in the subsequent paragraphs (See Figure 8). 

**Figure 9:**
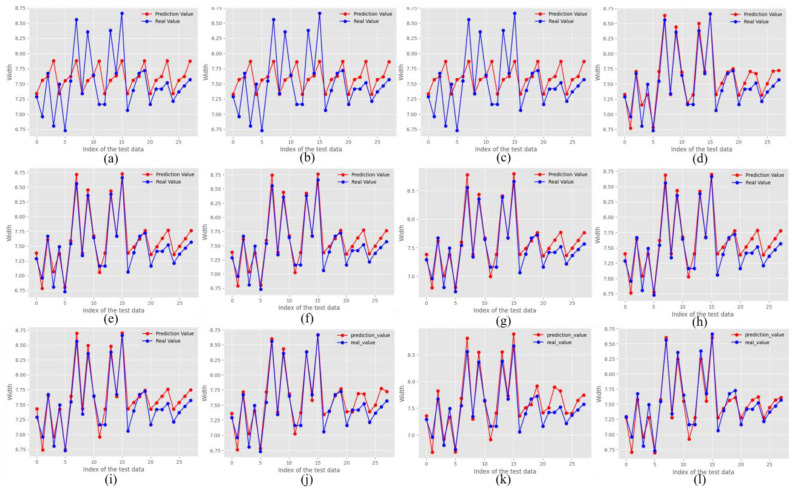
Predicted width vs. actual width (**a**) RF1, (**b**) RF2, (**c**) RF3, (**d**) RF best parameter, (**e**) Linear regression, (**f**) Polynomial (Quadratic) regression, (**g**) Polynomial (Cubic) regression, (**h**) Ridge regression, (**i**) Lasso regression, (**j**) S1, (**k**) S2, and (**l**) Proposed DNN model.

In the evaluation of width predictions, as mentioned earlier, the proposed DNN model demonstrated lower MAPE values than the RF and regression models. In addition, when considering the RMSE and $R^{2}$ values, the performance of the DNN model provided a more distinct picture. The first stacking model, which also showed competitive performance, had RMSE values close to those of the DNN model, indicating its effectiveness in capturing the overall data patterns. The $R^{2}$ values, which measure the proportion of variance in the dependent variable that is predictable from the independent variables, suggest that the DNN model can explain a significant portion of the variance in width predictions (See Figure 9).

**Figure 10:**
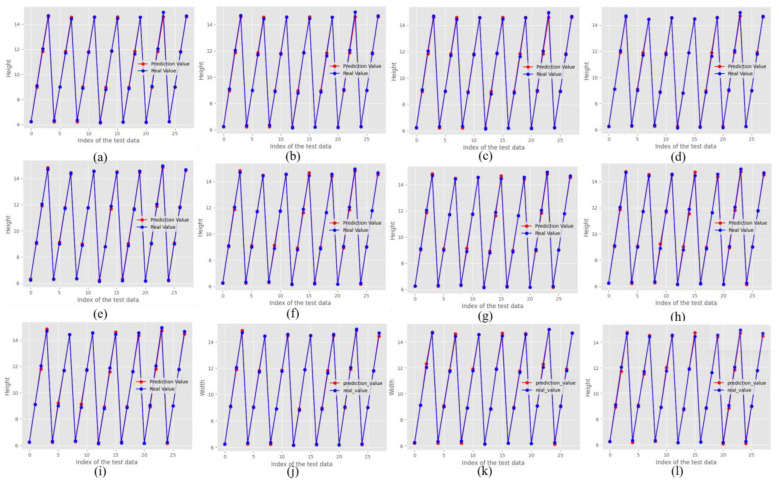
Predicted height vs. actual height (**a**) RF1, (**b**) RF2, (**c**) RF3, (**d**) RF best parameter, (**e**) Linear regression, (**f**) Polynomial (Quadratic) regression, (**g**) Polynomial (Cubic) regression, (**h**) Ridge regression, (**i**) Lasso regression, (**j**) S1, (**k**) S2, and (**l**) Proposed DNN model.

For the height predictions in Figure 10, the line plots illustrate a closer alignment between the real and predicted values across all models, with the DNN model consistently maintaining a competitive performance. The RF models, particularly RF3, exhibited lower prediction errors, highlighting their robustness and ability to capture complex relationships within the bead geometry data. This lower error in the RF models can be attributed to their ensemble learning approach, which reduces the risk of overfitting by averaging multiple decision trees. Similarly, the first stacking model showed strong alignment with the actual height predictions, suggesting that it effectively captured the height dimensions. Regression models such as Ridge Regression (RR) and Lasso Regression (LasR) also perform well, indicating their effectiveness in handling the variability of a given dataset through regularization techniques that penalize large coefficients, thereby preventing overfitting. 

The RF models with their ensemble approach managed to reduce overfitting, leading to lower prediction errors and demonstrating their robustness in practical scenarios. By contrast, linear regression models, which are simpler and easier to interpret, have limitations in capturing the non-linear relationships inherent in the data, resulting in less accurate predictions than more complex models. The second stacking model, although showing slightly higher errors in width prediction, maintained a strong performance in height prediction, underscoring its effectiveness. Overall, the competitive performance of the DNN model highlights its potential for applications that require a balance between accuracy and generalization. However, there is still room for optimization to enhance its precision, which could involve tuning the hyperparameters, increasing the depth and complexity of the network, or incorporating more advanced techniques such as dropout to prevent overfitting.

**Figure 11:**
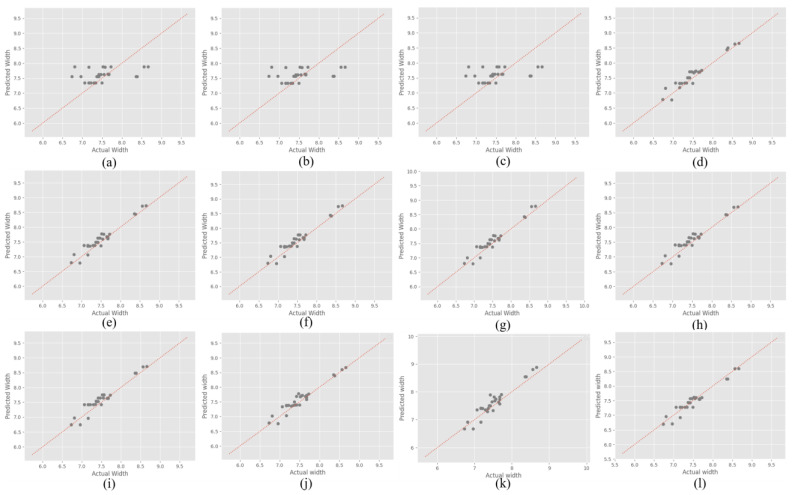
$R^{2}$ scatter plot of width (**a**) RF1, (**b**) RF2, (**c**) RF3, (**d**) RF best parameter, (**e**) Linear regression, (**f**) Polynomial (Quadratic) regression, (**g**) Polynomial (Cubic) regression, (**h**) Ridge regression, (**i**) Lasso regression, (**j**) S1, (**k**) S2, and (**l**) Proposed DNN model.

The $R^{2}$ analysis, as depicted in Figure 11, highlights the performance of the proposed DNN model compared with the RF and regression models in predicting the bead width. The DNN model exhibits a strong correlation between the predicted and actual bead widths, as evidenced by the data points (gray circles) clustering closely along the line of perfect prediction (dotted red line), with an $R^{2}$ value of 0.932. In contrast, the RF models show varying degrees of scatter, with the GSRF performing better than the other RF configurations but still falling short of the DNN’s performance. The $R^{2}$ values of the RF models were generally low, indicating less accurate predictions.

Among the regression models, cubic and quadratic regressions exhibited better performance, closely following the line of perfect prediction, particularly for height predictions. However, the DNN model surpasses them, particularly in capturing the full range of data variability, as indicated by its high $R^{2}$ values for both width and height. The linear and ridge regression models showed noticeable deviations from the ideal line, suggesting that they were less effective in modeling the complexities of the dataset. The scatter plots collectively demonstrate that while traditional models, such as RF and Polynomial regressions, perform well, the DNN model offers superior predictive accuracy and correlation, effectively capturing the nuances of the bead geometry data.

**Figure 12:**
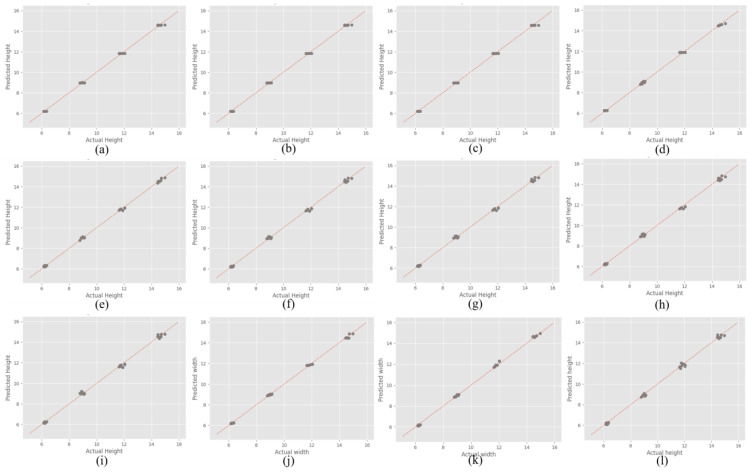
$R^{2}$ scatter plot of height (**a**) RF1, (**b**) RF2, (**c**) RF3, (**d**) RF best parameter, (**e**) Linear regression, (**f**) Polynomial (Quadratic) regression, (**g**) Polynomial (Cubic) regression, (**h**) Ridge regression, (**i**) Lasso regression, (**j**) S1, (**k**) S2, and (**l**) Proposed DNN model.

In Figure 12, the scatter plot compares the $R^{2}$ values of the height prediction for the models, where the predicted versus actual height reveals a close alignment along the diagonal line (indicating perfect prediction) for most models. The proposed DNN model exhibited the strongest performance, closely matching the actual heights, suggesting a high $R^{2}$ value, which is indicative of an excellent model fit and predictive accuracy. This was followed by the grid-searched best-parameter model among the Random Forest models, which also demonstrated robust predictive capability, although with slightly more deviation from the diagonal line compared with the DNN model. The first Stacking model also showed a strong fit, particularly for height prediction, highlighting the effective use of multiple models to achieve high accuracy. Among the regression models, cubic regression shows the best fit, outperforming Linear, Quadratic, Ridge, and Lasso regressions, but still falls short of the accuracy of the DNN. The scatter plots of the regression models indicate varying degrees of dispersion around the diagonal line, with Linear and Lasso regressions showing the most considerable deviation, suggesting lower $R^{2}$ values.

## 6. Conclusions

In conclusion, this study demonstrated the significant potential of DNN to predict bead geometry in the WAAM process, specifically focusing on the GMAW-CMT WAAM method. The DNN model outperformed the traditional regression models and Random Forest in predicting the bead width and height, achieving notable accuracy. Furthermore, the Stacking models, particularly the configuration combining Random Forest and Gradient Boosting, showed competitive performance, particularly in height prediction, where they achieved an accuracy similar to that of the DNN model. This advancement not only enhances the precision of the WAAM process but also contributes to the broader application of deep learning in manufacturing. There are several promising directions for the expansion of this study. One area of focus is the prediction of surface roughness, which is crucial for the quality and performance of manufactured parts. By integrating the surface roughness prediction model with the bead geometry prediction model, we aim to develop a reinforcement learning framework that optimizes surface roughness [34]. Implementing reinforcement learning algorithms can provide an adaptive approach to optimizing the bead geometry and surface roughness by continuously learning from the manufacturing process. However, further refinement will be needed to address limitations such as variations in material properties, real-time environmental changes, and computational efficiency in large-scale industrial applications. In practical terms, this framework can be applied in industries such as aerospace and automotive manufacturing, where precision and surface quality are critical. Additionally, integrating image-based analysis of melt pool fluid dynamics can further refine prediction models and capture real-time variations in the behavior of the melt pool.

Another exciting avenue for future research is the development of physics-driven models, such as physics-informed DNNs and CNNs. These models can leverage the fundamental physical laws governing melt pool behavior, providing more accurate and interpretable predictions. Physically s-driven DNNs and CNNs, such as temperature gradients, fluid flow, and solidification patterns, can be particularly useful for characterizing complex phenomena in the melt pool. Moreover, exploring further stacking configurations or other ensemble methods can enhance the model robustness and accuracy, particularly in scenarios involving complex data patterns or varying process parameters. This approach could lead to more robust models that not only predict outcomes, but also provide insights into the underlying physical processes. Moreover, expanding the dataset to include a broader range of materials, process parameters, and environmental conditions would enhance the generalizability of the model. In summary, this study lays the groundwork for more sophisticated and comprehensive modeling techniques for WAAM. By incorporating advanced deep learning methods, physics-based approaches, and real-time data analytics, future research can continue to improve the accuracy and efficiency of additive manufacturing processes, paving the way for innovative applications in a variety of industries.

## Figures and Tables

**Table 1:** Parameters for the GMWA-CMT WAAM process.

Parameters		Unit	Values
Dynamic process parameters	Travel speed	cm/min	1–12,000
	Feed rate	cm/min	100–1000
Static process parameters	Previous layer temperature	°C	100
	Arc length (bead to arc distance)	mm	5
	Wire diameter	mm	1.2
	Wire feeding angle	Degree	30
	Shielding gas	%	100
	Flow rate	L/min	20

**Table 2:** Wire feed rate and travel speed combination for the design of experiments.

Combination No.	Feed Rate (cm/min)	Travel Speed (cm/min)
1	480	30
2	560	31
3	650	33

**Table 3:** Structure of input (features) and output (labels/targets) of the DNN model.

			Input Data/Features				Output Data/Targets/Labels (Bead Shape of Next Layer)	
			Bead Shape of Current Layer		Parameter for Next Layer			
Index	# of Thin Wall	Layer	Width (mm)	Height (mm)	Travel Speed (cm/min)	Feed Rate (cm/min)	Width (mm)	Height (mm)
1	1	1st	7.645	3.327	30	480	6.959	5.41
2	1	2nd	6.959	5.41	30	480	6.91	9.14
⋮	⋮	⋮	⋮	⋮	⋮	⋮	⋮	⋮
107	27	3rd	7.366	9.017	31	560	7.467	11.785
108	27	4th	7.467	11.785	31	560	7.569	14.681

**Table 4:** Performance comparison of the proposed model with respect to the ML model.

Model	Model Parameters							Result (Width, Height)	
Regression	Degree							MAPE (%)	RMSE
1	Linear (LR)							(0.017, 0.008)	(0.148, 0.095)
2	Quadratic Polynomial (QR)							(0.018, 0.009)	(0.148, 0.097)
3	Cubic Polynomial (CR)							(0.018, 0.007)	(0.160, 0.084)
4	Ridge (RR)							(0.018, 0.010)	(0.156, 0.138)
5	Lasso (LasR)							(0.019, 0.012)	(0.164, 0.155)
Random Forest	N_etimators	Min_samples_leaf		Min_samples_split		Max_depth		MAPE (%)	RMSE
RF1	200	8		8		8		(0.043, 0.010)	(0.450, 0.130)
RF2	100	8		8		4		(0.043, 0.010)	(0.449, 0.130)
RF3	100	4		8		2		(0.043, 0.010)	(0.450, 0.132)
Grid Search (GSRF)	100	1		2		4		(0.016, 0.009)	(0.148, 0.109)
Stacking	Model 1			Model 2				MAPE (%)	RMSE
S1	Random Forest			Gradient Boosting				(0.016, 0.008)	(0.148, 0.096)
S2	XGBoost			CatBoost				(0.023, 0.009)	(0.199, 0.114)
DNN	Activation Function	Layer	Drop Out	Weight Initialization	Optimizer	Learning Rate	Epoch	MAPE (%)	RMSE
Proposed Model	Relu	(2, 8, 16, 32, 64, 128, 256, 128, 64, 32, 16, 8, 2)	None	He initialization	Adam	1 × 10^−4^	10,000	(0.014, 0.012)	(0.122, 0.153)

## Data Availability

Data are contained within the article.
